# Integrin-Linked Kinase Is Involved In the Proliferation and Invasion of Esophageal Squamous Cell Carcinoma

**DOI:** 10.7150/jca.33737

**Published:** 2020-01-01

**Authors:** Zhonghua Ning, Xiaozhong Zhu, Youqin Jiang, Aidi Gao, Shitao Zou, Chao Gu, Chao He, Yihong Chen, Wei-Qun Ding, Jundong Zhou

**Affiliations:** 1 Department of Radiation Oncology, The Third Affiliated Hospital of Soochow University, Changzhou, Jiangsu, P.R. China.; 2 Department of Thoracic Surgery, the Affiliated Hospital of the Jiangsu University, Zhenjiang, Jiangsu, P.R. China.; 3 Department of Radiation Oncology, The Third People's Hospital of Yancheng, Yancheng, Jiangsu, P.R. China.; 4 Suzhou Cancer Center Core Laboratory, Nanjing Medical University Affiliated Suzhou Hospital, Suzhou, Jiangsu, P.R. China.; 5 Department of Gastrointestinal surgery, Nanjing Medical University Affiliated Suzhou Hospital, Suzhou, Jiangsu, P.R. China.; 6 Department of Radio-Oncology, The First Affiliated Hospital of Wannan Medical College, Wuhu, Anhui, P.R. China.; 7 Department of Pathology, University of Oklahoma Health Science Center, Oklahoma City, OK, USA.

**Keywords:** Integrin-linked kinase, Proteomics, Esophageal squamous cell carcinoma, Metastasis, Tumor invasion.

## Abstract

Esophageal squamous cell carcinoma (ESCC) is an aggressive type of cancer with high mortality rate in China, largely due to its high invasive and metastatic potential. The purposes of this study are to investigate the potential molecular mechanisms behind the aggressive nature of ESCC and search for new prognostic biomarkers. By employing the quantitative proteomic based strategy, we compared the proteomic profile between three ESCC samples and paired adjacent tissues. After bioinformatics analysis, four candidate proteins were validated in thirteen paired patient samples. Further validation of the key candidate, integrin-linked kinase (ILK), was carried out in one hundred patient samples. The specific inhibitor compound 22 (cpd22) was used to assess the influence of ILK to ESCC cell motility and invasiveness by applying wound-healing and transwell assay. Western blot analysis was performed to elucidate the signaling pathways involved in ILK-mediated ESCC invasion. Total 236 proteins were identified by proteomic analysis. Bioinformatics analysis suggested a key role of the collagen/integrin/ILK signaling pathway during ESCC progression. Further validation indicated that ILK is overexpressed in ESCC tissues and is correlated with poor patient prognosis. Inhibition of ILK kinase activity suppresses proliferation and blocks invasion and migration of ESCC cells. Signaling pathway analysis revealed that ILK regulates AKT phosphorylation on Ser473 but not GSK-3β on Ser9 to promote proliferation and motility of ESCC cells. In conclusion, our results indicated that ILK may play a crucial role in ESCC invasion and metastasis and may serve as a prognostic biomarker and therapeutic target for ESCC.

## Introduction

Esophageal cancer is one of the fatal malignant tumors in China with only 15% to 25% of 5-year survival rate 1, 2. In recent years, the incidence is increasing and more than 90% of esophageal cancer in China is esophageal squamous cell carcinoma (ESCC). At present, surgical treatment is the main therapeutic strategy for esophageal cancer, followed by radiotherapy and chemotherapy. Since metastasis and invasion of ESCC occur in the early stage, and symptoms are more hidden, specificity is not high, easy to ignore. Therefore, many patients are found to be in the advanced stage, the surgical effect is poor or has lost the opportunity to operate 3. Early detection, diagnosis and treatment are the key to improve the efficacy and long-term survival. Tumor molecular biology studies have shown that the occurrence of tumor is closely related to cell proliferation and differentiation disorders, and tumor invasion and metastasis is the main biological characteristics, affecting its therapeutic effect and prognosis 4. Therefore, it is very important to find the genes closely related to the occurrence, development, metastasis and invasion of esophageal cancer for early diagnosis, targeted therapy and prognosis observation.

Integrin-linked kinase (ILK) is a serine/threonine protein kinase which binds to cytoplasm domain of integrin β1 and distribute in the cytoplasm near the membrane. It has dual effects in the process of carcinogenesis, progression and metastasis. Firstly, ILK regulates multiple signal transduction pathways, including AKT, GSK-3β, Nf-κB, Erk and β-catenin signaling pathway 5-9. Secondly, ILK forms a scaffold complex with some cytoskeleton proteins and plays a crucial role in regulating cell motility, cytoskeleton reorganization, focal adhesion, tumor progression and invasion 10. Recently, evidences suggested that, ILK was overexpressed in many cancers and contributed to proliferation, invasion and metastasis 11-13. However, ILK expression and its role in regulating ESCC cell proliferation and invasion remained obscure.

In this study, a quantitative proteomic based strategy was employed to investigate differential expressed proteins between ESCC tissues and adjacent normal tissues. Bioinformatics analysis suggested that ILK expression was elevated in ESCC tissues. Further validation indicated that expression of ILK was positively correlated with poor prognosis. Inhibition of ILK kinase activity by specific inhibitor termed Compound 22 (cpd22) 14 blocked ESCC cells proliferation, invasion and migration and attenuated AKT phosphorylation. Thus, investigating the role of ILK in ESCC would provide a significant improvement in diagnosis and treatment.

## Materials and Methods

### Tissue samples and cell lines

Human ESCC tissues and adjacent tissues used in this study were obtained from Nanjing Medical University Affiliated Suzhou Hospital (Jiangsu, China). The tissue samples were immediately snap-frozen and stored at -80°C. All of the samples were obtained with informed consent and the study was approved by the Institutional Ethics Committee of Nanjing Medical University. Human ESCC cell lines, including ECA-109 and KYSE-150, as well as human esophageal epithelial cell line HEEC-1, were obtained from Shanghai Cell Bank (Shanghai, China). ECA-109 cells were cultured in DMEM medium, KYSE-150 cells and HEEC-1 cells were cultured in RPMI-1640 medium. All of the media (Hyclone, Logan, UT, USA) were supplemented with 10% FBS (Hyclone). The cells were incubated in a humidified atmosphere, with 5% CO_2_ at 37°C.

### Experimental design

To explore potential candidates for therapeutic targets and prognostic biomarkers, a TMT-labeled quantitative proteomic analysis of paired ESCC (group A) and adjacent normal tissues (group B) was performed. ESCC tissues with lymph node metastasis were analyzed, including two stage III tissues and one stage II-b tissue. Grouped tissue samples were pooled and labeled with 6-plex TMT reagents of different mass (samples from group A were labeled by 126-128 and group B were labeled by 129-131). Thirteen paired tissue samples were included for validation of four selected protein candidates using immunohistochemistry. For survival curve analysis, one hundred of ESCC tissues and eighty paired adjacent normal tissues were applied. For molecular mechanism study, both ECA-109 and KYSE-150 cells were treated with the specific ILK inhibitor compound 22 (cpd22, Millipore, Billerica, MA, USA). Cpd22 was dissolved in DMSO for stock solution, and diluted in culture medium at appropriate concentrations. After treatment, cell viability, migration and invasion assays were performed.

### Protein extraction and trypsin digestion

Sample was grinded by liquid nitrogen and sonicated three times on ice using a high intensity ultrasonic processor (Scientz, Ningbo, China) in lysis buffer containing 8 M urea, 1% Triton-100, 65 mM DTT and 0.1% Protease Inhibitor Cocktail (Sigma-Aldrich, St. Louis, MO, USA). The remaining debris was removed by centrifugation at 20,000 g at 4 °C for 10 min. The protein was precipitated with cold 15% TCA for 2 h at -20 °C. The precipitant was washed with cold acetone for three times. The protein was redissolved in the buffer (8 M urea, 100 mM TEAB, pH 8.0) and the protein concentration was determined with 2-D Quant kit (GE Healthcare, Piscataway, NJ, USA) according to the manufacturer's instructions. Before digestion, the protein solution was reduced with 10 mM DTT (Sigma-Aldrich) for 1 h at 37 °C and alkylated with 20 mM IAA(Sigma-Aldrich) for 45 min at room temperature in darkness. For trypsin digestion, the protein sample was diluted by adding 100 mM TEAB to urea concentration less than 2M. Finally, trypsin (Sigma-Aldrich) was added at 1:50 trypsin-to-protein mass ratio for the first digestion overnight and 1:100 trypsin-to-protein mass ratio for a second 4 h-digestion. Approximately 100 μg protein for each sample was digested with trypsin for the following experiments.

### TMT labeling and HPLC fractionation

After trypsin digestion, peptide was desalted by Strata X C18 SPE column (Phenomenex, Torrance, CA, USA) and vacuum-dried. Peptide was reconstituted in 0.5 M TEAB and processed according to the manufacturer's protocol for 6-plex TMT kit (Thermo Scientific, Rochester, NY, USA). The 6 samples were mixed and then fractionated into fractions by high pH reverse-phase HPLC using Agilent 300Extend C18 column (5 μm particles, 4.6 mm ID, 250 mm length, Agilent, Santa Clara, CA, USA). The peptides were combined into 18 fractions and dried by vacuum centrifuging.

### LC-MS/MS and bioinformatics analysis

Peptides were dissolved in 0.1% FA (Sigma-Aldrich), directly loaded onto a reversed-phase pre-column (Acclaim PepMap 100, Thermo Scientific). The resulting peptides were analyzed by Q Exactive^TM^ hybrid quadrupole-Orbitrap mass spectrometer (Thermo Scientific). LC-MS/MS analysis, database search and bioinformatics annotation were performed by PTM-Biolabs Co., Ltd (Hang Zhou, China). Gene Ontology and KEGG pathway analysis and visualization were performed using R (Version 3.5.0) with the package clusterProfiler (Version 3.8.1) 15. Protein-protein interaction networks were predicted by STRING. Network visualization was performed by Cytoscape (Version 3.4.0). Genes were considered to be significantly differentially expressed between groups when the *P*-value was less than 0.05 and the fold change of expression was more than 2.0.

### Immunohistochemical analysis

Tumor tissue sections were deparaffinized and heat-treated with citrate buffer, pH 6.0, for 5 min as an epitope retrieval protocol. The tissue sections were then exposed to 0.03% hydrogen peroxide for 5 min to block endogenous peroxidase activity followed by incubation with 2% BSA to block nonspecific antibody binding sites. Then the tissue sections were incubated with ITGA1, ITGB1, VCL and ILK antibodies (Abcam, Cambridge, MA, USA) at proper concentration according to manufacturer's instructions for 2 h at 37°C. HRP-conjugated anti-mouse/rabbit antibody (Multi Science, Hangzhou, China) was then added for 1 h and the color was developed using 3-30-diaminobenzidine. Following washing step, the sections were counterstained with hematoxylin, washed and dipped briefly in a water bath containing drops of ammonia, prior to dehydration and mounting in Diatex. The stained sections were analyzed and scored using a Leica Microscope (Leica, Wetzlar, Germany). Scoring was based on the intensity and average percentage of positive cells. The staining intensity was scored with “1” (negative or weakly positive), “2” (moderately positive) and “3” (strongly positive). The average percentage of positive cells was scored as: 1 (<25%), 2 (25-50%), 3 (50-75%) and 4 (>75%).

### Cell viability assay

ECA-109, KYSE-150 and HEEC-1cells were seeded in 96-well plate at 4,000 cells per well. After 24 h, cells were treated with the specific ILK inhibitor compound 22 for 24 h. Cell viability was determined using CCK-8 cell counting kit (Multi Sciences) according to manufacturer's instructions. 10 μL of CCK-8 was added to each well and incubated for 3 h. Absorbance was measured at 450 nm.

### Wound-healing and transwell assays

For wound-healing assay, ECA-109, KYSE-150 and HEEC-1cells were seeded in six-well plates and reached confluence in 24 h. A thin mark was drawn vertically with a pipette tip in the six-well plate. Cells were then washed three times with PBS to remove the floating and detached cells. Fresh serum-free medium containing cpd22 at the concentration of 3 μM, 1.5 μM and 1 μM for ECA-109, KYSE-150 and HEEC-1 were added, and photos were taken at an appropriate time to assess cell migration using a light microscope (Leica Corporation).

Cell invasion was assessed using Matrigel-coated Transwell chambers (Corning, NY, USA). Cells were plated in medium without FBS in 24-well Transwell inserts pre-coated with Matrigel at 1:8 dilution (BD Bioscience, Billerica, MA, USA). Cpd22 was added at the concentration identical to wound-healing assay. The lower chambers were filled with medium that contained 20% FBS. After incubation for 24 or 48 h, the cells remaining in the upper chambers were scraped off, and the invading cells were stained with Wright-Giemsa solution (Nanjing Jiancheng Bioengineering Technology, Nanjing, China). The penetration of cells through the membrane was photographed under a microscope.

### Western blot analysis

Cells were harvested and lysed in RIPA lysis buffer (Beyotime Biotechnology, Shanghai, China) containing protease and phosphatase inhibitors (Beyotime Biotechnology) for 20 min at 4°C. Equal amounts of the proteins were separated by SurePAGEᵀᴹ precast polyacrylamide gels with a gradient between 4-20% (GenScript, Nanjing, China) and transferred to PVDF membranes (Millipore). After blocking with 5% nonfat milk (BioRad, Hercules, CA, USA), the membranes were incubated with primary antibodies against β-actin, AKT, phospho-AKT (S473), phospho-AKT (T308), GSK-3β, phospho-GSK-3β (S9) (Multi Science) and ILK (Abcam). The membranes were then incubated with an HRP-conjugated anti-mouse or anti-rabbit secondary antibody (Multi Sciences). The protein bands were visualized using High-sig ECL Western Blotting Substrate (Tanon, Shanghai, China). Images were collected using the Tanon-5200 Chemiluminescent Imaging System (Tanon). β-actin protein expression was detected as loading control for each sample.

### Statistical analysis

The quantitative results are presented as the mean values ± SEM. Statistical analyses were performed using SPSS 19.0 software (IBM, Chicago, IL, USA). Statistical significance was considered at a *P*-value of <0.05. Differences between groups were estimated using the χ2-test and Student's t-test. The overall survival rate was calculated actuarially according to the Kaplan-Meier method and analyzed by the log-rank test. Relationships of variables were explored using Pearson's correlation.

## Results

### Differential proteomics analysis of ESCC and adjacent tissues

**Figure 1:**
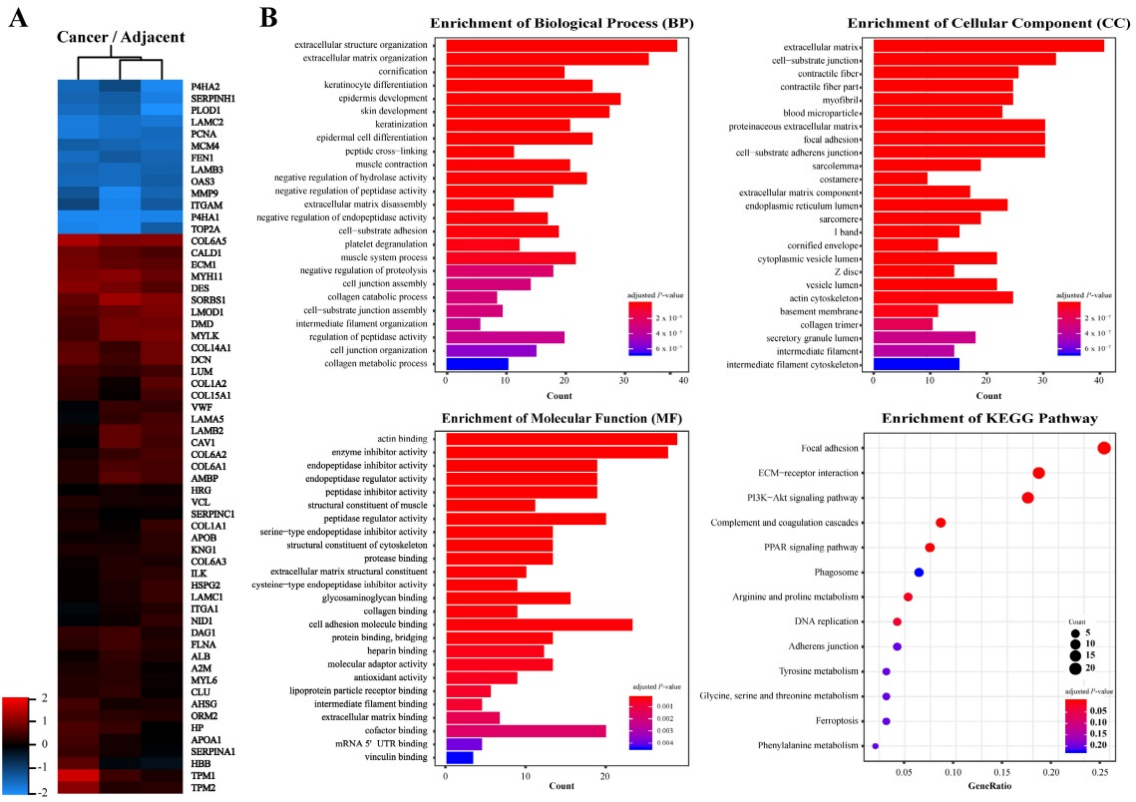
** Proteins identified from ESCC and adjacent normal tissues by LC-MS/MS.** A, Heat map of differentially expressed proteins. B, Gene ontology (GO) analysis and KEGG pathway analysis of differentially expressed proteins. Terms of GO analysis and KEGG pathway analysis were marked with a color gradient from red to blue and ranked by *P*-values. The bar length of each GO terms and sphere size of each KEGG terms represents gene counts enriched, respectively.

In order to identify key molecules that regulate ESCC invasion and metastasis, quantitative proteomics was applied to analyze the protein profiles of ESCC and adjacent tissues. Proteins with a fold change higher than 2.0 and *P*-value less than 0.05 were considered significantly differentially expressed. A total of 236 proteins were identified in ESCC tissues, of which expression of 73 was down-regulated and 163 was up-regulated as compared to adjacent tissues (Fig. 1A, Table S1). Gene Ontology annotations suggested that the Biological Processes involved in differentially expressed proteins include: extracellular structure organization, extracellular matrix organization; the Cellular Components include: extracellular matrix, cell-substrate junction; and the Molecular Function include: actin binding, enzyme inhibitor activity, extracellular matrix structural constituent, collagen binding (Fig. 1B). KEGG Pathway enrichment indicated that the pathways involved in differentially expressed proteins include: Focal adhesion, ECM-receptor interaction, and PI3K-Akt signaling (Fig. 1B). These results suggested that significantly differentially expressed proteins between ESCC and adjacent tissues are enriched in extracellular matrix and receptor-related signaling pathways.

### Protein interaction network analysis

**Figure 2:**
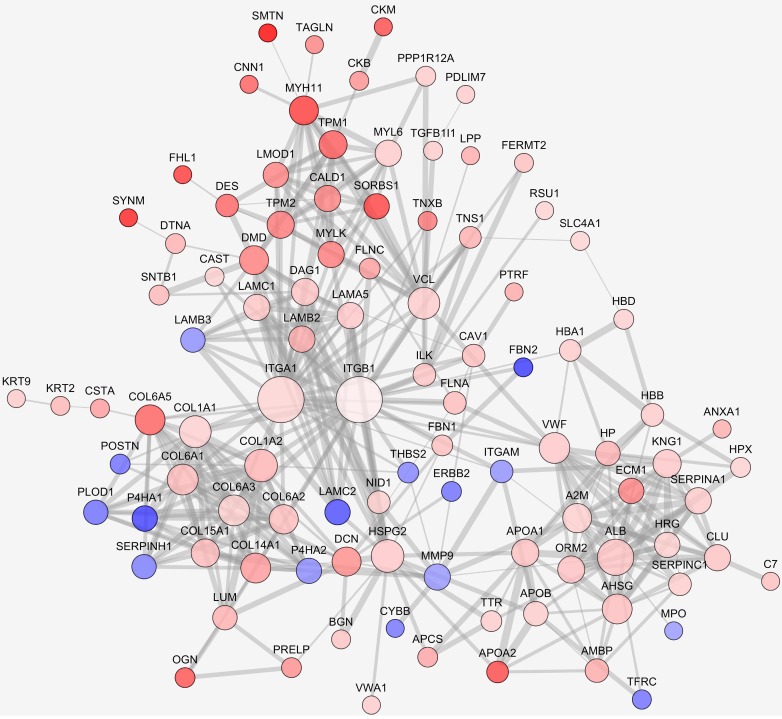
** Protein-protein interaction networks of differentially expressed proteins.** Protein interaction was predicted by STRING. Up- or down-regulated proteins were indicated in red or blue, respectively. The darker color indicates the higher fold change. The larger node represents more protein-protein interactions, the thicker line represents stronger interaction.

We further analyzed the interaction network of differentially expressed proteins. Interactions were predicted by STRING with interaction score higher than 0.7. The largest network consisted of 100 nodes was analyzed. As seen in Fig. 2, expression of many ECM related proteins was significantly changed, with the upregulation of several collagen family proteins being conspicuous. Although the fold change was less than 2.0, ITGB1 was singled out as an interesting candidate since the complex of ITGA1 and ITGB1 can be activated by collagen. In the network, ITGA1 and ITGB1 were located to the key node and most extensively participated in the interactions. Among the interacting participants, there were several proteins involved in cell-ECM communication and actin remodeling, such as ILK, vinculin (VCL), myosin light chain kinase (MYLK), filamin A (FLNA) and filamin C (FLNC). Based on the network analysis, a potential collagen/integrin α1β1/ILK signaling pathway was envisioned. In addition, a previous study suggested that VCL is involved in cell-ECM adhesion, integrin signaling and actin remodeling 16. Thus, ITGA1, ITGB1, ILK and VCL were selected as candidate proteins for further validation.

### Validation of candidate proteins

**Figure 3:**
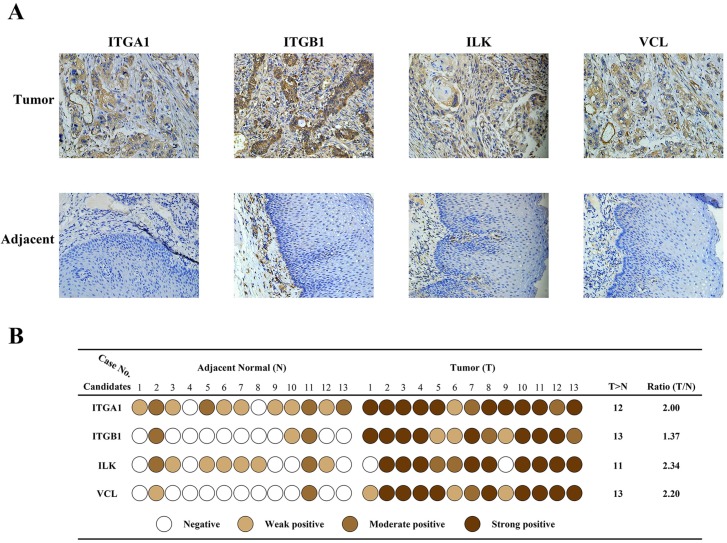
** Validation of differentially expressed protein**. A, Immunohistochemical staining of four protein candidates in ESCC and adjacent normal tissues. B, Diagram of immunohistochemical staining in all thirteen pairs of patient tissues. The IHC score of each tissue was converted into one of four levels based on the intensity and percentage of staining, and was represented by different colors. The ratio indicates the fold change identified by proteomics.

Immunohistochemistry was carried out in 13 paired specimens to validate expression of the four candidate proteins (Fig.3A). The IHC score was classified in four grades: negative, weak positive, moderate positive, strong positive, based on the staining intensity and percentage of positive cells. As shown in Fig.3B, scores of each candidate in each specimen were represented by different colors. The Ratio column represents Fold change ratio of differential proteomics. Results indicated that all four candidates were highly expressed in cancer tissues, consistent with proteomics results. In adjacent tissues, expression of ITGA1 and ILK were weak or moderate positive, and expression of ITGB1 and VCL were negative or weak positive. These results confirmed that the expression of the four candidates was elevated in cancer tissues. Considering the key transducer role of ILK in cell-matrix communication and integrin signaling, we focused on ILK for further investigation.

### Expression of ILK was elevated in cancer tissues and was correlated with poor prognosis

**Fig 4:**
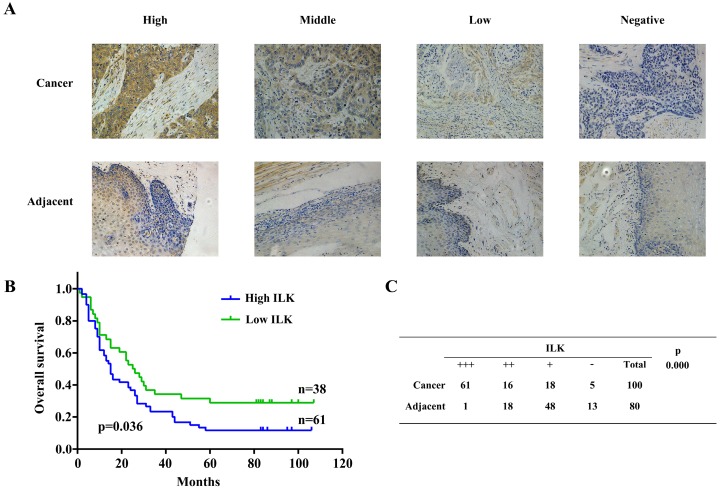
** ILK was overexpressed in ESCC tissues and correlated with poor prognosis.** A, Immunohistochemical staining of ILK in ESCC and adjacent normal tissues. B, Kaplan-Meier survival analysis based on expression of ILK. C, Expression of ILK in ESCC and adjacent normal tissues.

Further investigation was carried out using 100 ESCC samples, 80 of them were paired with adjacent tissues. Expression of ILK was detected by immunochemistry staining. IHC scores of ILK were classified identical to the previous validation step (Fig.4A). Next, we employed Kaplan-Meier survival analysis to evaluate the relationship between ILK expression and patient prognosis. Since one patient was lost to follow-up, 99 patients were divided into two groups based on their ILK expression scores. Patients with high ILK level had the survival rate less than 20%, whereas it was approximately 40% survival rate for patients with low ILK expression (Fig.4B). These results indicated that elevated ILK expression was correlated with poor prognosis. Subsequently, we evaluated ILK expression levels between cancer and adjacent tissues (Fig.4C). Again, ILK was found to be overexpressed in cancer tissues as compared to adjacent tissues. Similar results were observed in cell lines. As shown in Fig.S1, ILK was overexpressed in ESCC cell lines as compared to HEEC-1. These results suggested that ILK plays a key role in ESCC progression and is a potential prognostic biomarker for this malignancy.

### Inhibition of ILK activity suppressed proliferation and blocked invasion and metastasis of cancer cells

**Fig 5:**
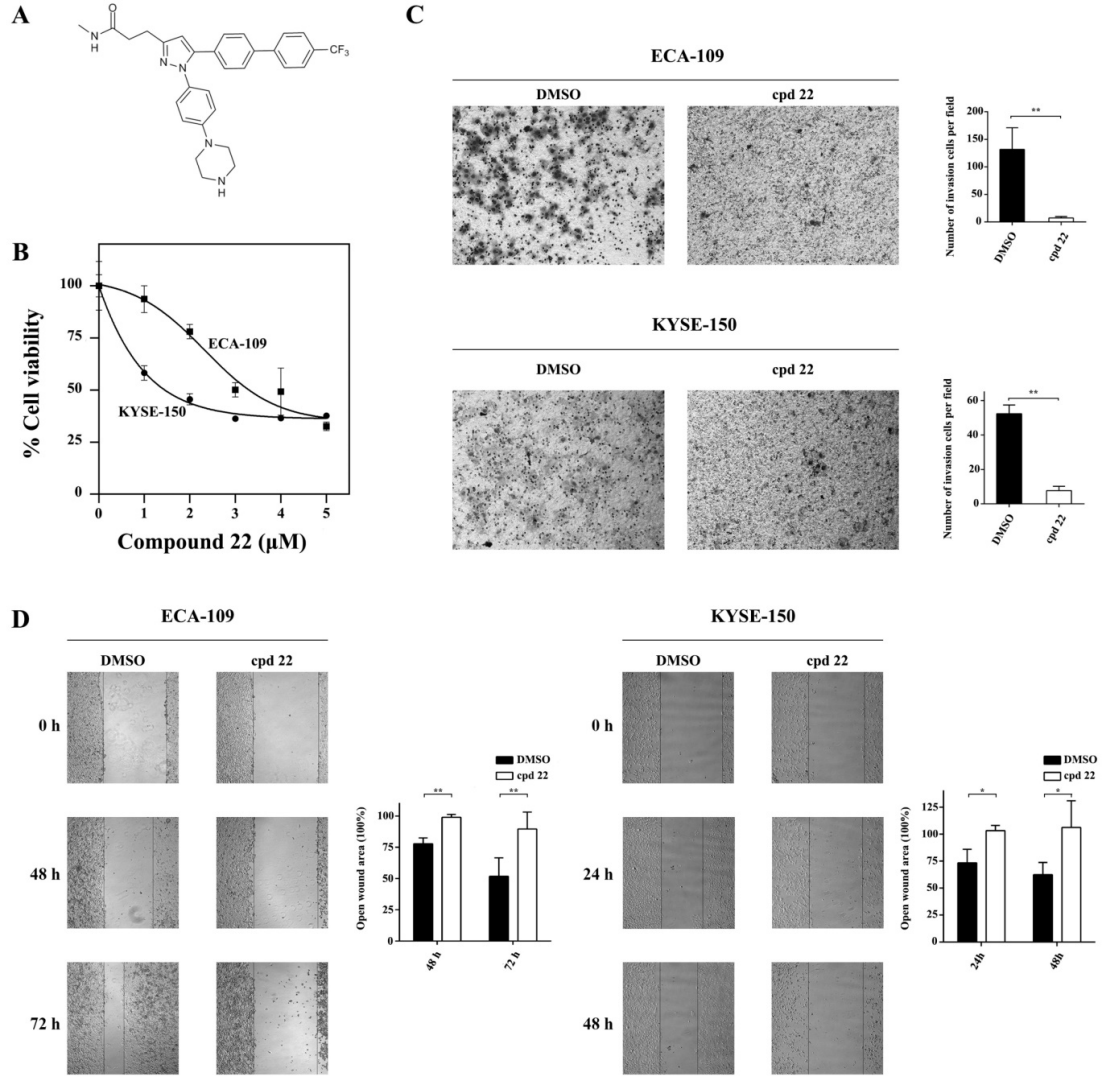
** Inhibition of ILK attenuated ESCC cell proliferation, invasion and migration.** A, Structure of ILK specific inhibitor Compound 22 (cpd22). B, Both ECA-109 and KYSE150 cells were treated with cpd22 for 24 h and cell viability was deternined by the CCK-8 cell counting kit. IC_50_ of ECA-109 and KYSE-150 cells are 3.5 μM and 2 μM, respectively. C, Transwell assay was performed to study the invasion of ECA-109 and KYSE-150 cells (n=3). 1 x 10^5^ cells were seeded per chamber. After cpd22 treatment for 48 h or 24 h, the cells were stained with Wright-Giemsa solution and photographed at x200. D, Wound-healing assay was applied to measure the migration of ECA-109 and KYSE-150 cells (n=5). Cells were seeded at a concentration of 3 x 10^5^ cells/mL per well in a 6-well plate and photographed under a microscope at x40. Student's t-test was employed to analyze the results, *P < 0.05, **P < 0.01.

A well-established ILK specific inhibitor, the compound 22 (cpd22, Fig.5A) was applied to suppress ILK kinase activity in ECA-109 and KYSE-150 cells. Cpd22 was previously identified by screening a compound library and proven to inhibit ILK's kinase activity 14. Cell viability assay indicated that cpd22 significantly inhibited proliferation of ESCC cells in a concentration-dependent manner (Fig.5B). IC_50_ for ECA-109 and KYSE-150 cells were 3.5 μM and 2 μM, respectively. For HEEC-1 cells, IC_50_ was 1.8 μM (Fig.S2A).

Since ILK played crucial role in cancer cell motility, we evaluated the effect of cpd22 on cancer cell invasion and metastasis. As expected, both ECA-109 and KYSE-150 cells treated with cpd22 failed to invade through matrigel-coated upper chamber, comparing to DMSO treated control cells (Fig.5C). Furthermore wound-healing assay indicated that the open wound area remains unchanged in cpd22 treated cells but is diminished to approximately half of the initial area in DMSO treated group (Fig.5D). Besides, the effect of cpd22 on HEEC-1 cells was assessed. Few cells were able to invade through the upper chamber in both groups (Fig.S2B). The open wound area in DMSO treated group diminished slightly, while it has remained unchanged in Compound 22 treated group (Fig.S2C). These findings demonstrated that kinase activity of ILK is crucial to the motility of ESCC cells.

### ILK suppressed proliferation and motility of ESCC cells through the AKT signaling pathway

**Fig 6:**
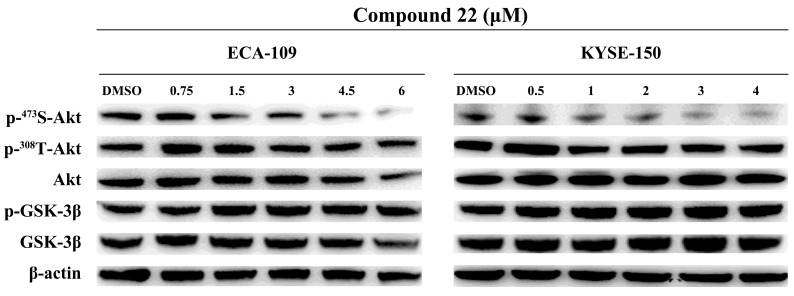
** Inhibition of ILK attenuated AKT phosphorylation in ESCC cells.** Phosphorylation level of AKT on Ser473 was decreased in a concentration-dependent manner after inhibiting ILK activity by cpd22. While phosphorylation level of GSK-3β on Ser9 remain unchanged.** Fig.1. Proteins identified from ESCC and adjacent normal tissues by LC-MS/MS.** A, Heat map of differentially expressed proteins. B, Gene ontology (GO) analysis and KEGG pathway analysis of differentially expressed proteins. Terms of GO analysis and KEGG pathway analysis were marked with a color gradient from red to blue and ranked by *P*-values. The bar length of each GO terms and sphere size of each KEGG terms represents gene counts enriched, respectively.

Having proven the critical role of ILK kinase activity in ESCC proliferation and motility, we further explored the downstream signaling pathways involved in these processes. The most important substrates of ILK were AKT and GSK-3B. Therefore, phosphorylation levels of AKT on Ser473 and Thr308, as well as GSK-3β on Ser9 were analyzed. ECA-109 and KYSE-150 cells were treated with a series of concentrations of cpd22 and the same amount of DMSO was added as vehicle control. As shown in Fig. 6, the phosphorylation level of AKT on Ser473 decreased in a concentration-dependent manner, while phosphorylation on Thr308 remained unchanged. Unexpectedly, inhibition of ILK kinase activity had no influence on GSK-3β on Ser9 phosphorylation. These results indicated that ILK facilitates ESCC cell proliferation and migration through the AKT signaling pathway by phosphorylation on Ser473 of AKT, rather than the GSK-3β pathway in our model systems.

## Discussion

In China, esophageal squamous cell carcinoma ranks the third of incidence and the forth cause of cancer-related death 1. It is a type of lethal cancer due to its high invasive and metastasis potential. Despite recent advances in esophageal cancer treatment, the prognosis of esophageal cancer remains poor. Thus, there is urgent need for screening new biomarker and therapeutic target. In our quantitative proteomic based study, a total of 236 differentially expressed proteins were identified. Among them, ILK was selected as key candidate based on the central signal transducing location in protein-protein interaction network and its key role in cancer progression and cancer cell motility.

It has been proven that overexpression of ILK was crucial for carcinogenesis and metastasis in assorted cancer types, including colorectal cancer 8, renal cell carcinoma 11, thyroid cancer 12, breast cancer 6, 13, non-small cell lung cancer 17, 18, prostate cancer 19, 20. In colorectal cancer cells, overexpression of ILK promoted proliferation, metastasis, and invasion ability. On the contrary, downregulation of ILK in breast cancer significantly inhibited tumorigenic and metastatic potential 13. In prostate cancer, the expression of ILK increased significantly with the progression of prostate cancer 20. Nevertheless, whether ILK contributes to ESCC carcinogenesis and progression is not well known. In our present study, it was confirmed that ILK expression was positive correlated with poor prognosis (*P* = 0.036). Simultaneously, ILK was remarkably overexpressed in ESCC tissues compared with adjacent tissues (*P* < 0.001). Thus, ILK may serve as a latent clinical biomarker for prognosis and distant metastasis.

Recently, ILK was reported to be a central regulator in ECM/integrin signaling pathway. On the one hand, ILK was reported to regulate AKT and GSK-3β signaling pathway by phosphorylating AKT on Ser473 and GSK-3β on Ser9, respectively 5, 9, 13, 14, 21, 22. Evidences suggested that both downregulated expression and inhibited kinase activity of ILK lead to attenuated phosphorylation of Ser473 and GSK-3β, resulting suppressed tumor growth and metastasis 14, 23-27. In order to investigate the mechanism of ILK in ESCC, we further explored the downstream signaling pathways. Likewise, our kinase inhibition study found that, in our circumstance, phosphorylation of AKT on Ser473 attenuated after treating with specific inhibitor cpd22 in a dose-depended manner, resulting in suppressed proliferation and blocked invasion of ESCC cells. These evidences indicate that ILK may play a key role in ESCC proliferation and invasion. Interestingly, the phosphorylation level of GSK-3β on Ser9 remained constant after cpd22 treatment. One possible explanation is that phosphorylation level of GSK-3β is regulated by other kinase, such as protein kinase A (PKA), p90 ribosomal S6 kinase/MAPK activating protein (p90RSK/MAPKAP) and p70 ribosomal S6 kinase (p70S6K) 28. On the other hand, ILK functioned as a scaffold protein to form a multiple protein complex with other important regulators of actin cytoskeleton 29, 30. In our study, GO annotation indicated that many differentially expressed proteins were located to actin cytoskeleton (cellular component); other proteins were found to have actin binding activity (molecular function). These results suggested that in ESCC tissues, ILK upregulation my contributed to actin remodeling process. Therefore, searching for ILK interacting partners may gain more insights into the mechanism of ILK in ESCC.

Taken together, our proteomic profiling demonstrates that overexpression of ILK activates AKT signaling cascade, result in ESCC proliferation, invasion and metastasis. Our findings provide new insight into ESCC carcinogenesis and progression, indicating that ILK may serve as a latent biomarker and therapeutic target.

## Supplementary Material

Supplementary figures.

Supplementary tables.

## Abbreviations

ILK: Integrin-Linked Kinase
ESCC: Esophageal Squamous Cell Carcinoma
cpd22: compound 22
AKT: AKT serine/threonine kinase
GSK-3β: glycogen synthase kinase 3β
Nf-κB: nuclear factor kappa B
Erk: extracellular signal-regulated kinases
ITGA1: integrin α1
ITGB1: integrin β1
VCL: vinculin
ECM: extracellular matrix
