# Supplementary figures and images for: Integrin-Linked Kinase Is Involved In the Proliferation and Invasion of Esophageal Squamous Cell Carcinoma
Source: J Cancer. 2020 Jan 1;11(2):324–33. doi: 10.7150/jca.33737 (PMC6930430; doi:10.7150/jca.33737)

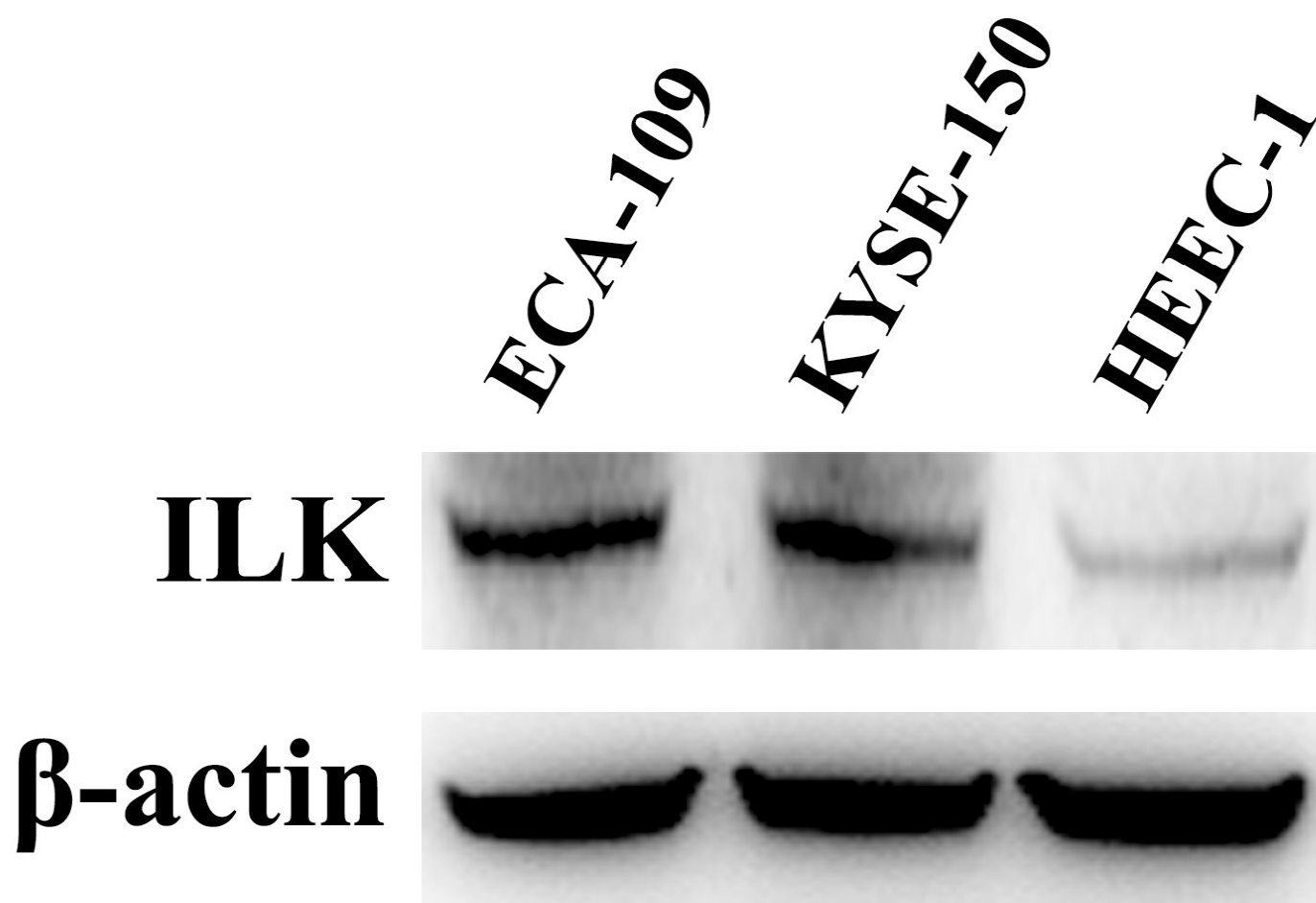

Fig.S1

**A**

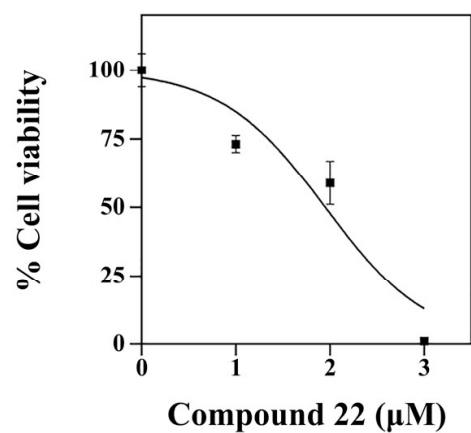

**B**

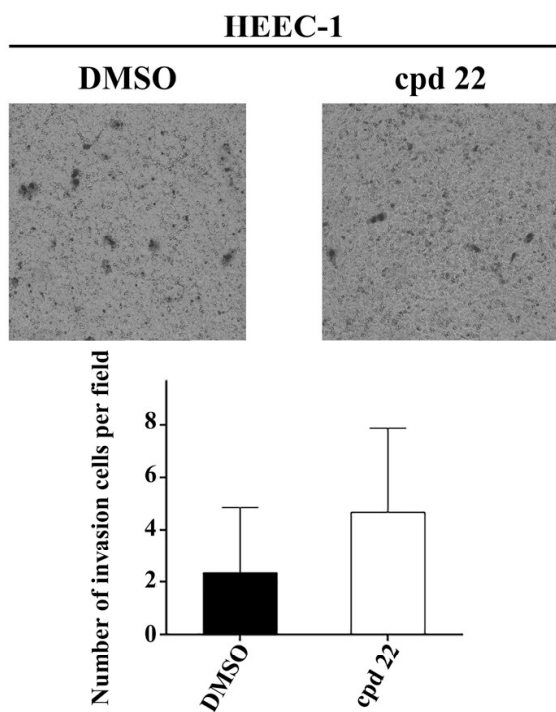

**C**

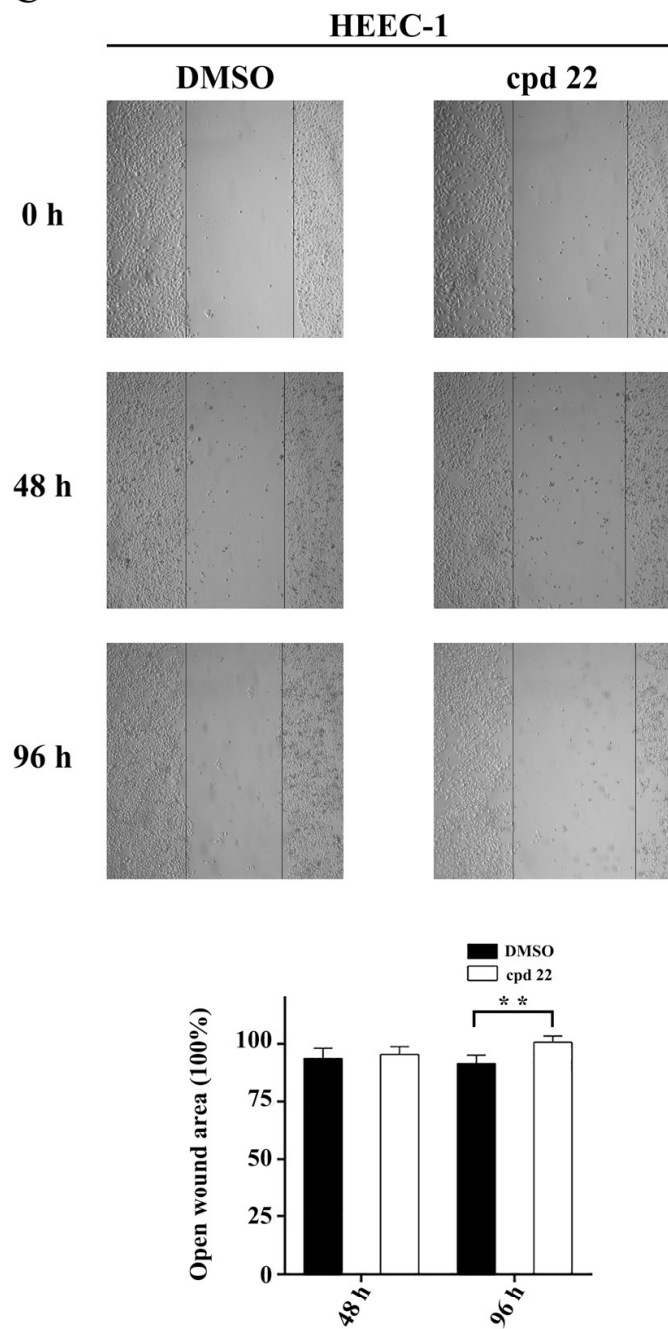

Fig.S2

Supplement: Supplementary file 1 — Supplementary figures. [file jcav11p0324s1.pdf]
